# Cisplatin and ε-Viniferin Synergistically Modulate Oxidative Stress in HeLa Cells: Implications for Redox Modulation in Cervical Cancer Cells

**DOI:** 10.3390/molecules31142409

**Published:** 2026-07-08

**Authors:** Tayyar Görkem Sayer, Gamze Yılmaz, Filiz Özdemir

**Affiliations:** 1 Department of Biochemistry, Faculty of Pharmacy, Anadolu University, 26470 Eskişehir, Türkiye; tgsayer@anadolu.edu.tr; 2 Department of Biochemistry, Graduate Education Institute, Anadolu University, 26470 Eskişehir, Türkiye; gamze580@anadolu.edu.tr

**Keywords:** ε-viniferin, cisplatin, HeLa cell, combination chemotherapy

## Abstract

This study investigates the combined effects of cisplatin (CDDP) and ε-viniferin (ε-VNF), a natural stilbenoid, on oxidative stress and apoptosis in HeLa cells. Cytotoxicity was assessed using the MTT assay, and IC_50_ values were determined as 28 µM for CDDP and 21 µM for ε-VNF. Synergistic and antagonistic combination ratios of these doses were tested. Oxidative stress was evaluated via Total Oxidant Status (TOS), Total Antioxidant Status (TAS), Oxidative Stress Index (OSI), Superoxide Dismutase (SOD), Reduced Glutathione (GSH), and Malondialdehyde (MDA). Apoptosis was measured using Annexin V-FITC/PI staining and caspase-9 activation assays. TAS levels significantly increased in all combination groups compared to the control (control: 266.7 ± 0.1 µmol/L; 20% combo: 2466.7 ± 1.0 µmol/L). OSI values decreased accordingly (control: 22.5 ± 7.1; 10% combo: 1.6 ± 0.5). GSH levels decreased in the combination groups (e.g., 20%: 0.8 ± 0.2 µM vs. control: 1.4 ± 0.1 µM), while MDA levels increased (20%: 3.8 ± 0.5 µM vs. control: 0.5 ± 0.1 µM). Caspase-9 positive cells increased markedly (20%: 55.0% vs. control: 13.2%), supporting activation of the mitochondrial apoptotic pathway. Annexin V analysis revealed increased late apoptosis (20%: 76.1%) and early apoptosis (20%: 17.0%).

## 1. Introduction

Cervical cancer is the fourth most common malignancy among women worldwide and continues to pose a significant global health burden, particularly in low- and middle-income countries [1]. Each year, over 500,000 new cases are diagnosed, with more than 300,000 deaths reported globally [2]. Persistent infection with high-risk human papillomavirus (HPV) types is the primary etiological factor. Although vaccination programs have been implemented in many regions, late-stage diagnosis and treatment resistance still limit clinical outcomes.

For advanced cervical cancer, treatment strategies include radiotherapy, chemotherapy, and, when necessary, surgical intervention. Cisplatin (cis-diamminedichloroplatinum(II), CDDP) remains a cornerstone chemotherapeutic agent in cervical cancer treatment and was the first metal-based compound approved for anticancer use [3,4,5]. However, its long-term clinical efficacy is often limited by the development of chemoresistance and cumulative toxicity. These limitations have prompted increasing interest in combination therapies that can enhance antitumor efficacy while minimizing adverse effects.

Oxidative stress is a key factor in carcinogenesis and cancer progression, resulting from an imbalance between the generation of reactive oxygen species (ROS) and cellular antioxidant defense mechanisms [6,7]. Depending on the concentration and cellular context, ROS may exhibit dual roles, acting as signaling molecules that promote proliferation or as cytotoxic agents that induce apoptosis. Numerous anticancer agents exert their therapeutic effects, at least in part, via ROS overproduction [8,9]. Dietary antioxidants and functional food components can support redox homeostasis by scavenging ROS, preventing oxidative damage, and modulating cellular signaling and gene expression pathways [10]. In cancer cells, elevated ROS levels may arise due to enhanced metabolic activity, mitochondrial dysfunction, oncogene activation, and altered redox signaling, leading to oxidative stress [11]. While normal cells possess adaptive mechanisms to counter oxidative insults, malignant cells are often more vulnerable due to their intrinsic oxidative burden, which contributes to genomic instability, aggressive tumor behavior, and altered sensitivity to anticancer drugs [12,13].

Natural products continue to play a pivotal role in drug discovery, with approximately 25–48% of FDA-approved anticancer agents being derived from plant-based sources [14]. These compounds can function as pro-oxidants or antioxidants depending on cellular context and may modulate multiple signaling pathways related to cell survival and death. Among these, stilbenoids represent a prominent group of polyphenolic compounds with a C6–C2–C6 structure, commonly found in various plant species such as *Vitis vinifera*, a primary source of resveratrol and its derivatives [15,16]. Resveratrol is known for its diverse biological activities and is synthesized by plants as a defense response against UV radiation, toxins, and microbial attack [17,18]. Viniferins (VNF), oligomeric derivatives of resveratrol, include several isomers such as α-, β-, δ-, ε-, and γ-viniferins [19]. While resveratrol exhibits poor bioavailability due to limited absorption, ε-viniferin (ε-VNF) shows significantly enhanced in vivo absorption and stability, making it a promising candidate for therapeutic development [20,21]. ε-VNF has been reported to exert various biological effects, including anti-inflammatory [22], antioxidant [23], anti-obesity [24], cardioprotective [25], and neuroprotective [26] activities. In this context, resveratrol and, in particular, its derivatives, such as ε-VNF, are considered promising natural agents for the treatment of various diseases, including cancer. In recent years, beyond its antioxidant and anti-inflammatory properties, the anticancer potential of ε-VNF has also attracted increasing scientific interest. Preclinical investigations in A549 cells and other cancer models, including osteosarcoma and non-small cell lung cancer, have demonstrated that epsilon-viniferin induces apoptosis, an effect mediated by decreased levels of phosphorylated Akt and increased expression of cleaved PARP and caspase-3. Based on these findings, the use of ε-VNF in combination with chemotherapeutic agents may contribute to the development of more effective and less toxic therapeutic strategies [27,28]. Despite increasing interest in natural polyphenols as anticancer agents, studies investigating the combined effects of ε-VNF and cisplatin in cervical cancer models remain scarce. To date, there is limited data on how ε-VNF may influence redox balance and apoptotic responses when used alongside conventional chemotherapy agents such as CDDP in cervical cancer.

In this study, we aimed to investigate the individual and combined effects of cisplatin and ε-viniferin on HeLa cervical cancer cells, focusing on oxidative stress markers [Total Oxidant Status (TOS), Total Antioxidant Status (TAS), Oxidative Stress Index (OSI), Superoxide Dismutase (SOD), Malondialdehyde (MDA), Reduced Glutathione (GSH)] and apoptotic indicators (Annexin V-FITC/PI, caspase-9). We hypothesized that ε-VNF may enhance the anticancer efficacy of cisplatin by modulating redox homeostasis and promoting apoptosis, offering a potential novel strategy for cervical cancer treatment.

## 2. Results and Discussion

Nowadays, due to changes in dietary habits, sedentary lifestyles, and various genetic predispositions, cancer has become a prevalent and potentially fatal disease [29]. Although chemotherapy has demonstrated significant success in cancer treatment, it is also associated with severe adverse effects in patients. Therefore, there is an increasing need for the development of new therapeutic strategies that can be used either in addition to or as an alternative to conventional chemotherapy. In recent years, the promising therapeutic potential of natural compounds in the treatment of various diseases has also gained attention in the field of cancer therapy. In this context, the present study aimed to investigate the combination effects of ε-VNF, a natural compound, with CDDP, a well-known chemotherapeutic agent, on HeLa cells. Specifically, the effects of this combination on oxidative stress and apoptosis were evaluated.

### 2.1. Cell Viability Effects of CDDP and ε-VNF on HeLa Cells

**Figure 1:**
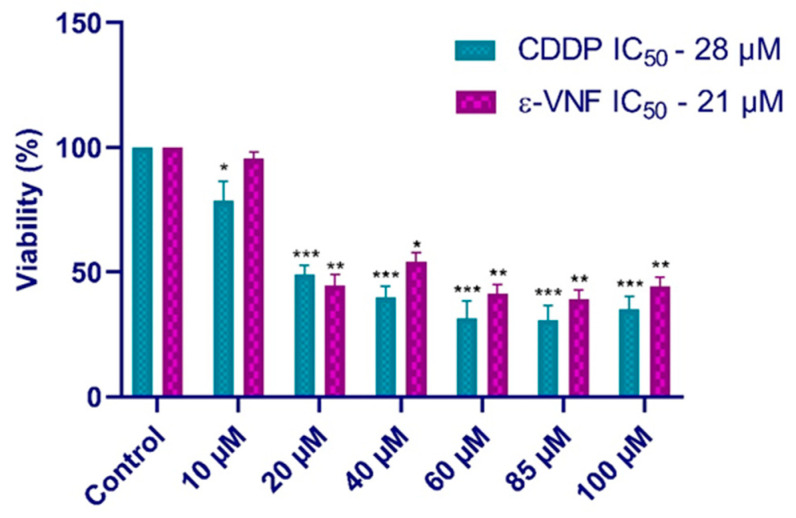
Dose-dependent effects of CDDP and ε-VNF on HeLa cell viability after 24 h of treatment, as measured by the MTT assay. A significant decrease in cell viability was observed with increasing concentrations of both agents. CDDP exhibited a cytotoxic effect starting at 10 µM, while ε-VNF showed a significant reduction in viability beginning at 20 µM. IC_50_ values were calculated as 28 µM for CDDP and 21 µM for ε-VNF. Data are presented as mean ± SD of three independent experiments. * *p* < 0.05, ** *p* < 0.01, *** *p* < 0.001 versus control.

The cytotoxic effects of CDDP and ε-VNF on HeLa cells were evaluated using the MTT [3-(4,5-dimethylthiazol-2-yl)-2,5-diphenyltetrazolium bromide] assay after 24 h of treatment with increasing concentrations (10–100 µM). As shown in Figure 1, both agents caused a concentration-dependent decrease in cell viability. Notably, CDDP significantly reduced cell viability starting from 10 µM (* *p* < 0.05), whereas ε-VNF exhibited a significant effect beginning at 20 µM (** *p* < 0.01). The half-maximal inhibitory concentration (IC_50_) values were calculated as 28 µM for CDDP and 21 µM for ε-VNF, suggesting that ε-VNF may exhibit higher cytotoxic potency in HeLa cells (Table 1). At higher concentrations (≥60 µM), both compounds appeared to reach a plateau phase in cytotoxicity, with no substantial further reduction in viability, indicating a saturation of effect. These results support the potential of both agents as effective inhibitors of HeLa cell proliferation and provide a basis for evaluating their combined effects in subsequent experiments.

### 2.2. Effect of CDDP and ε-VNF Combination on HeLa Cell Viability

**Figure 2:**
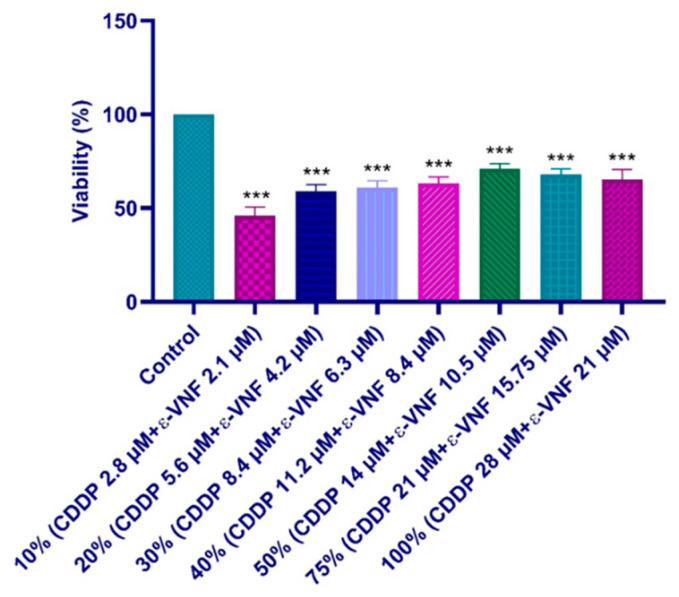
Combined effects of CDDP and ε-VNF on HeLa cell viability after 24 h of treatment. Cells were treated with increasing percentages (10–100%) of the IC_50_ doses of CDDP (28 µM) and ε-VNF (21 µM) in a fixed 1:1 ratio. Cell viability was assessed using the MTT assay. A significant reduction in viability was observed across all combination groups compared to the untreated control. The most pronounced effect was observed at 10% of IC_50_ concentrations. Data are presented as mean ± SD of three independent experiments. *** *p* < 0.001, statistically significant compared to the control group.

To evaluate the combined effects of CDDP and ε-VNF on cell viability, HeLa cells were treated with serial dilutions (10–100%) of their IC_50_ concentrations, maintaining a 1:1 ratio. As shown in Figure 2, all combination groups significantly reduced cell viability compared to the control group (*** *p* < 0.001). Notably, the lowest concentration tested (10% of IC_50_; CDDP: 2.8 µM + ε-VNF: 2.1 µM) resulted in a marked decrease in viability, greater than that observed with higher single-agent doses. Increasing the combination dose did not result in a proportionally greater cytotoxic effect, suggesting that the maximal inhibitory response was achieved at low concentrations. These findings imply a possible synergistic interaction between CDDP and ε-VNF, which may allow for dose reduction while maintaining therapeutic efficacy.

### 2.3. Evaluation of Combination Index (CI)

**Figure 3:**
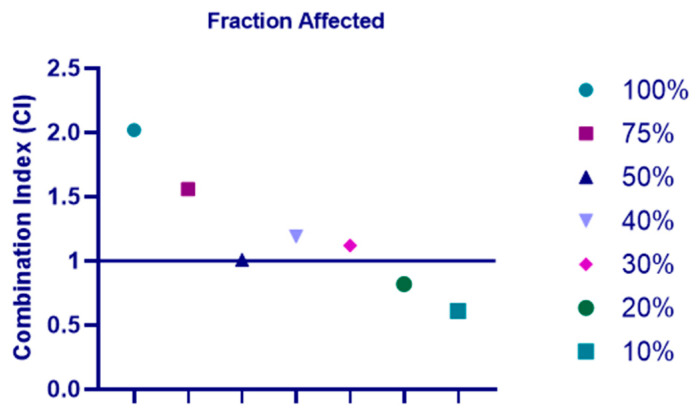
CI values of ε-VNF and CDDP at various effect levels (Fa: 10–100%) are shown. CI values were calculated using the Chou–Talalay method, where CI > 1 indicates antagonism, CI = 1 indicates an additive effect, and CI < 1 indicates synergism. At higher effect levels (75–100%), the combination exhibited antagonistic interactions; at 50%, an additive effect; and at lower effect levels (10–30%), a synergistic interaction was observed. These results suggest that the combination may be more effective at lower doses. *Fa (fraction affected): the proportion of inhibited or affected cells in response to treatment*.

To assess the interaction between CDDP and ε-VNF, Combination Index (CI) values were calculated using CompuSyn software based on the Chou–Talalay method. As shown in Figure 3, the combination exhibited a dose-dependent shift in interaction profile. Strong synergistic effects were observed at the lowest combination doses, particularly at 10% and 20% of the IC_50_ doses (CI ≈ 0.55 and 0.75, respectively). Moderate to weak synergism or additivity was noted at 30–40%, while higher dose combinations (≥75%) demonstrated antagonistic interactions (CI > 1). These findings indicate that low-dose combinations may provide maximal cytotoxic effect with reduced drug burden, and support the rationale for further investigation of low-dose regimens. Based on MTT assay results, the IC_50_ values of CDDP and ε-VNF on HeLa cells were determined to be 28 µM and 21 µM, respectively (Table 1; Figure 1). These values are consistent with the CDDP IC_50_ value of 22.4 µM for HeLa cells reported by Becit et al. In the same study, the combination of CDDP and curcumin significantly reduced the viability of both HeLa and HepG2 cells [30]. Similarly, extracts derived from *Vitis vinifera* species have also been reported to suppress the growth of these cancer cell lines [31]. In our study, ε-VNF alone exhibited low cytotoxicity; however, its combination treatment with CDDP, particularly at 10% and 20% ratios, led to a marked decrease in cell viability, indicating a synergistic effect. In contrast, the 40% combination group showed an antagonistic response. These findings are in agreement with the study by Huang et al., which demonstrated that lower-dose combinations of α-VNF and ε-VNF produced stronger synergistic effects compared to single-agent treatments in A549 cells [32]. Based on these results, the 10% and 20% synergistic combinations and the 40% antagonistic group were selected for further evaluation of their effects on oxidative stress and apoptosis.

### 2.4. Oxidative Balance Status

#### 2.4.1. Total Oxidant Status/Total Antioxidant Status Levels

Changes in Total Oxidant Status (TOS) and Total Antioxidant Status (TAS) levels according to CDDP and ε-VNF IC_50_ doses and combination groups in HeLa cells are shown in Table 2. A statistically significant decrease in TOS levels was observed in HeLa cells treated with CDDP and ε-VNF IC_50_ doses (31.7 ± 4.4 and 20.0 ± 5.0 µmol/L, respectively), compared to the control group (61.7 ± 9.3 µmol/L). Similar reductions were also detected in the 40% (35.0 ± 1.7 µmol/L) and 10% (21.7 ± 4.4 µmol/L) combination groups. No statistically significant change was observed in the 20% combination group (43.3 ± 6.0 µmol/L). Although TAS levels were also elevated in the IC_50_ dose and other combination groups (CDDP: 666.7 ± 0.1, ε-VNF: 1133.0 ± 0.1, 10%: 1333.3 ± 0.1, 40%: 1266.7 ± 0.1 µmol/L), these increases were not statistically significant. The anticancer effect of CDDP primarily arises from its ability to form covalent adducts between platinum and DNA bases, thereby disrupting replication and transcription processes [33]. However, depending on its intracellular accumulation and exposure duration, it can also enhance the production of ROS [34]. While this oxidative stress contributes to therapeutic efficacy, it may also cause severe adverse effects such as nausea, vomiting, ototoxicity, neurotoxicity, myelosuppression, and notably, irreversible nephrotoxicity in approximately one-third of patients [35,36]. Therefore, novel therapeutic strategies are needed to reduce systemic toxicity while preserving the anticancer potency of CDDP. According to our results, TAS levels were elevated in all combination groups compared to the control group, although statistical significance was observed only in the 20% combination group. The 20% combination group showed the highest TAS value (2466.7 ± 1.0 µmol/L), followed by the 10% and 40% groups, whereas the TAS level in the control group was 266.7 ± 0.1 µmol/L. This increase may reflect a compensatory activation of cellular antioxidant defense mechanisms in response to elevated oxidative stress. OSI values, calculated as the TOS-to-TAS ratio, were also significantly reduced in all treatment groups, suggesting a shift toward antioxidant dominance (Table 3).

#### 2.4.2. Oxidative Stress Index

The Oxidative Stress Index (OSI) was calculated based on the measured TOS and TAS levels in HeLa cells. As shown in Table 3, all treatment groups exhibited significantly reduced OSI values compared to the control group (22.5 ± 7.1 µmol/L). The OSI values for the CDDP and ε-VNF IC_50_ doses were 5.0 ± 1.3 µmol/L and 1.7 ± 0.3 µmol/L, respectively (*** *p* < 0.001). Similar statistically significant reductions were observed in the combination groups: 40% (3.0 ± 2.1 µmol/L), 20% (1.8 ± 1.9 µmol/L), and 10% (1.6 ± 0.5 µmol/L) (*** *p* < 0.001 for all). These findings suggest that both single-agent and combination treatments effectively attenuate oxidative stress in HeLa cells, with the lowest OSI levels observed in the ε-VNF and 10% combination groups. Specifically, OSI values in the 10% and 20% groups were measured at 1.6 ± 0.5 and 1.8 ± 1.9, respectively, compared to 22.5 ± 7.1 in the control. Since TAS encompasses both enzymatic [e.g., Superoxide Dismutase (SOD), Catalase (CAT), Glutathione Peroxidase (GPx)] and non-enzymatic (e.g., glutathione, vitamins, uric acid) antioxidant components, it serves as a reliable marker for total antioxidant capacity [37]. Collectively, these results indicate that ε-VNF may reduce the oxidative burden induced by CDDP and support endogenous antioxidant responses.

**Table 3:** OSI values measured in HeLa cells after 24 h treatment with CDDP, ε-VNF at IC_50_ doses, and various fixed-ratio combinations. All treatment groups showed statistically significant decreases in OSI levels compared to the control group. Data are presented as mean ± SD from three independent experiments. *** *p* < 0.001, statistically significant compared to the control group.

Groups	OSI
Control	22.5 ± 7.1
CDDP IC_50_	5.0 ± 1.3 ***
ε-VNF IC_50_	1.7 ± 0.3 ***
40% (CDDP 11.2 µM + ε-VNF 8.4 µM)	3.0 ± 2.1 ***
20% (CDDP 5.6 µM + ε-VNF 4.2 µM)	1.8 ± 1.9 ***
10% (CDDP 2.8 µM + ε-VNF 2.1 µM)	1.6 ± 0.5 ***

### 2.5. Superoxide Dismutase Activity

SOD enzyme activity was measured in HeLa cells treated with CDDP, ε-VNF, and their combinations to evaluate the antioxidant response. As shown in Table 4, a statistically significant decrease in SOD activity was observed in the CDDP (0.5 ± 0.1 U/mL) and ε-VNF (0.4 ± 0.1 U/mL) IC_50_ groups, compared to the control (1.6 ± 0.1 U/mL) (* *p* < 0.05). Similarly, a significant reduction was observed in the 10% combination group (0.8 ± 0.1 U/mL). Interestingly, the 40% combination group exhibited a marked increase in SOD activity (1.8 ± 0.4 U/mL), exceeding even the control level, whereas the 20% group showed a moderate but non-significant change (1.1 ± 0.2 U/mL). These results suggest that low-dose treatments may suppress SOD activity, while higher combination doses may induce a compensatory antioxidant response.

The enzymatic antioxidant defense system mainly includes enzymes such as SODs, GPx, thioredoxin reductase, and CAT. These enzymes interact with their substrates and convert hydroperoxides into non-radical, inactive species, thereby protecting cells from oxidative damage. Among these, SODs are ubiquitously expressed across organisms and represent the first line of enzymatic defense against oxidative stress [38]. In our study, significantly higher SOD activity was observed in the 40% combination group compared to the control. Although this may seem contradictory at first glance due to the antagonistic effect observed in the MTT assay, it is biologically plausible. It is possible that ε-VNF attenuated the cytotoxic effects of CDDP in this group, allowing cells to survive and develop a compensatory antioxidant response by upregulating SOD production. In contrast, SOD activity was lower than control levels in the 10% and 20% combination groups, suggesting that alternative antioxidant mechanisms—such as the glutathione system or broader non-enzymatic networks reflected by TAS—may have played a more dominant role. This reduction may also reflect enzyme depletion due to excessive ROS production.

### 2.6. Reduced Glutathione Levels

Reduced Glutathione (GSH) levels in HeLa cells were measured to assess the intracellular antioxidant capacity following treatment. As shown in Table 5, significant reductions in GSH levels were observed in the CDDP IC_50_ (0.4 ± 0.2 µM; ** *p* < 0.01) and ε-VNF IC_50_ (0.6 ± 0.2 µM; * *p* < 0.05) groups compared to the control (1.4 ± 0.1 µM). A significant decrease was also observed in the 10% combination group (0.9 ± 0.1 µM; ** *p* < 0.01). However, no significant change was observed in the 20% and 40% combination groups (0.8 ± 0.2 and 1.4 ± 0.3 µM, respectively), with the 40% group showing GSH levels close to control. These findings suggest that high-dose combination treatment may mitigate glutathione depletion induced by single-agent therapies.

Elevated intracellular levels of GSH are one of the hallmarks of many cancer types, distinguishing cancer cells from healthy tissue due to their enhanced resistance to oxidative stress [39]. Conversely, GSH depletion is a well-established indicator of apoptosis progression, especially in response to pro-apoptotic stimuli [40]. This depletion is known to occur through oxidation of GSH to glutathione disulfide or conjugation with highly reactive compounds such as xenobiotics, chemotherapeutic agents, and transition metals [41,42]. In our study, GSH levels in the 10%, 20%, and 40% CDDP+ε-VNF combination groups were significantly lower than those in the control group. This suggests that the cells were exposed to oxidative stress and that their antioxidant reserves were depleted. As reported in previous studies, such GSH depletion may sensitize cells to ROS-mediated cell death. The simultaneous decrease in GSH levels and marked elevation in TAS values may initially appear contradictory. However, TAS reflects the cumulative activity of multiple enzymatic and non-enzymatic antioxidant systems rather than GSH alone. Thus, depletion of intracellular GSH may have triggered upregulation of alternative antioxidant pathways, including SOD, catalase, glutathione peroxidase, thioredoxin-related systems, and other redox-regulating molecules. In this context, the pronounced TAS elevation observed particularly in the 20% combination group may represent an adaptive cellular response to excessive oxidative stress induced by the combined treatment.

### 2.7. Malondialdehyde Levels

Malondialdehyde (MDA) levels, a marker of lipid peroxidation, were measured to evaluate the oxidative stress induced by ε-VNF and CDDP treatments. In the control group, the MDA level was detected as 0.5 ± 0.1 µM (Table 6). Treatment with CDDP at IC_50_ significantly increased the MDA level to 1.6 ± 0.1 µM, indicating that cisplatin induces notable oxidative stress. ε-VNF at IC_50_ also elevated MDA levels (0.9 ± 0.2 µM) compared to the control, but to a lesser extent than CDDP. In the combination groups, MDA levels increased markedly in a dose-dependent manner. The highest level was observed in the 40% combination group (CDDP 11.2 µM + ε-VNF 8.4 µM), reaching 4.3 ± 0.6 µM, which was found to be highly significant (*** *p* < 0.001). Similarly, the 20% combination group (CDDP 5.6 µM + ε-VNF 4.2 µM) showed a significant increase to 3.8 ± 0.5 µM (** *p* < 0.01). The 10% combination group also exhibited an elevated MDA level (3.4 ± 0.4 µM), although statistical significance was not specified. These findings suggest that the combination of ε-VNF and CDDP leads to greater lipid peroxidation compared to single-agent treatments, likely due to a synergistic enhancement of oxidative stress. This dose-dependent increase may contribute to enhanced membrane damage, potentially triggering cell death pathways such as apoptosis or ferroptosis.

To support this observation, we also measured MDA levels, a widely used marker of lipid peroxidation. MDA is a reactive dicarbonyl compound formed during the peroxidation of polyunsaturated fatty acids and reflects membrane lipid damage [43]. According to our results, MDA levels were significantly elevated in the 10%, 20%, and 40% combination groups compared not only to the control but also to the single-agent CDDP and ε-VNF groups. This increase suggests that low-dose CDDP, when administered with ε-VNF, enhances oxidative effects and increases lipid peroxidation. This finding is consistent with literature reporting that CDDP-induced ROS can modify cellular enzymes and structural proteins, thereby facilitating apoptotic pathways [44]. Lipid peroxidation and the formation of reactive intermediates can disrupt membrane fluidity and permeability, compromising cellular integrity and promoting apoptosis [45,46].

### 2.8. Apoptosis

#### 2.8.1. Annexin V-FITC/PI

**Figure 4:**
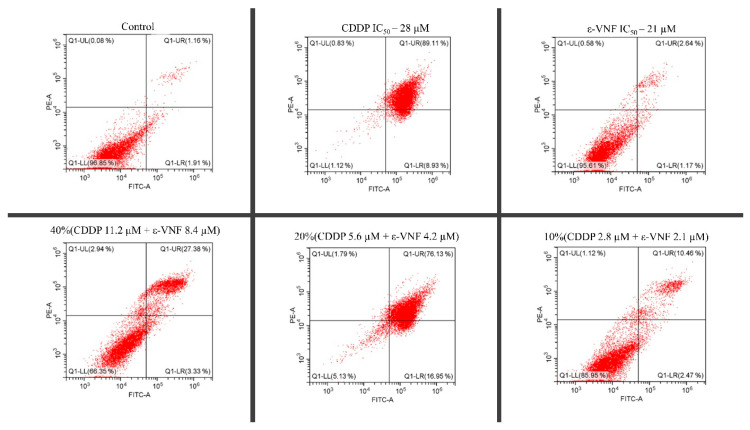
Flow cytometry analysis of apoptosis in HeLa cells after 24 h treatment with CDDP (28 µM), ε-VNF (21 µM), and their combinations. Apoptosis was assessed using Annexin V-FITC and propidium iodide (PI) staining. Each quadrant represents a specific cell population: Q1-LL: viable cells (Annexin V^−^/PI^−^), Q1-LR: early apoptotic cells (Annexin V^+^/PI^−^), Q1-UR: late apoptotic cells (Annexin V^+^/PI^+^), Q1-UL: necrotic cells (Annexin V^−^/PI^+^).

Apoptosis was assessed in HeLa cells using Annexin V-FITC/PI staining. As shown in Table 7 and Figure 4, treatment with CDDP IC_50_ resulted in a dramatic reduction in viable cells (1.1%) and a dominant late apoptotic population (89.1%), whereas ε-VNF IC_50_ alone had minimal impact, with 95.6% viability. Among the combination groups, the 20% combination induced the strongest apoptotic effect, reducing viability to 5.1%, with 17.0% early and 76.1% late apoptosis. The 40% combination preserved 66.4% viability but still triggered 27.4% late apoptosis. In contrast, the 10% combination caused minimal apoptotic response (86.0% viability). These results suggest that CDDP is the primary driver of apoptosis in this model, and its effect is retained or enhanced in specific combination ratios (notably 20%).

#### 2.8.2. Caspase-9 Activity

**Figure 5:**
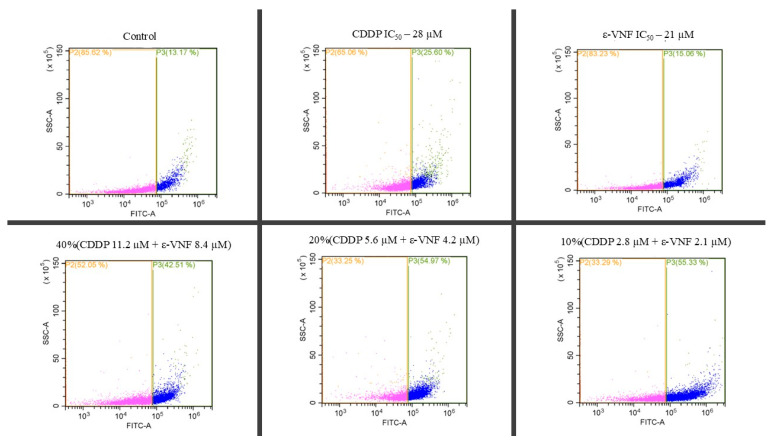
Flow cytometry analysis of active caspase-9 in HeLa cells after 24 h treatment with CDDP (28 µM), ε-VNF (21 µM), and their combinations at 10%, 20%, and 40% IC_50_ ratios. Caspase-9 activation was assessed using a FITC-conjugated caspase-9 detection kit. The percentage of caspase-9 positive cells (P3 region, right of the vertical marker) is indicated in blue, while caspase-9 negative cells are shown in pink (P2 region, left of the marker).

Caspase-9 activity was measured in HeLa cells after 24 h treatment to evaluate activation of the intrinsic apoptotic pathway. As shown in Table 8 and Figure 5, CDDP IC_50_ induced a moderate increase in caspase-9 positive cells (25.7%) compared to the control (13.2%). ε-VNF IC_50_ alone showed a minimal change (15.1%). In contrast, all combination groups demonstrated greater caspase-9 activation than single-agent treatments. Notably, the 10% and 20% combinations exhibited the highest levels of caspase-9 positive cells (55.3% and 55.0%, respectively), indicating strong activation of mitochondrial apoptosis. The 40% combination also showed considerable activation (42.5%).

At the core of this process lies the intrinsic (mitochondrial) apoptosis pathway, initiated by mitochondrial dysfunction. This mechanism is triggered by excessive ROS production during cellular respiration, which leads to the release of cytochrome c and the formation of the apoptosome complex with Apaf-1 and procaspase-9, resulting in the activation of caspase-9 and subsequent executioner caspases (e.g., caspase-3) [47,48]. In our study, these biochemical and mechanistic events were confirmed by Annexin V-FITC/PI flow cytometry and caspase-9 activity assays. Apoptotic responses in the combination groups were significantly higher compared to the control and single-agent groups. Collectively, these findings indicate that ε-VNF enhances oxidative stress and apoptotic responses when used in combination with CDDP, likely by amplifying mitochondrial dysfunction and intrinsic apoptotic signaling.

A study by Nessa et al. also reported that a combination treatment in which resveratrol was administered two hours prior to CDDP and oxaliplatin sensitized ovarian cancer cells to platinum-induced apoptosis [49]. Additionally, our previous study demonstrated that the CDDP + ε-VNF combination induced strong apoptotic effects in glioma cell lines (C6) [50].

In our study, the combination of CDDP and ε-VNF markedly increased caspase-9 activity, with the proportion of caspase-9 positive cells detected as 55.3%, 55.0%, and 42.5% in the 10%, 20%, and 40% combination groups, respectively. Compared to the control group (13.2%), these values strongly suggest that the applied treatment primarily triggered apoptosis via the mitochondrial pathway. This mechanism differs from the effects of resveratrol. For instance, a study by Zhang et al. reported that resveratrol induced apoptosis in HeLa cells through caspase-3 activation, but independently of caspase-9 activity and mitochondrial membrane potential [51].

Other studies have also highlighted functional differences between ε-VNF and resveratrol. In a study using U266 multiple myeloma cells, ε-VNF was shown to induce apoptosis by arresting the cell cycle at the G2/M phase, while resveratrol-treated cells accumulated in the S phase. Both agents triggered caspase-dependent apoptosis through disruption of mitochondrial membrane potential [52]. Furthermore, another study in prostate cancer cells emphasized that ε-VNF was significantly more effective than resveratrol in inhibiting cell proliferation and was the only compound shown to enhance apoptosis-related enzymatic activity [53].

Our findings were further supported by Annexin V-FITC/PI staining, which revealed significantly increased levels of apoptosis in the combination groups. Specifically, in the 20% combination group, 76.1% of cells were in late apoptosis and 17.0% in early apoptosis, while in the 10% group these rates were 10.5% and 2.5%, respectively. In contrast, the control group showed only 1.2% and 1.9% of cells in late and early apoptosis, respectively. These results phenotypically confirm the activation of the caspase-9-dependent apoptotic pathway.

Biochemical data associated with oxidative stress were also consistent with these findings. Notably, in the 20% combination group, a decrease in GSH levels (0.84 ± 0.2 µM), an increase in MDA levels (3.8 ± 0.5 µM), and changes in SOD activity (1.1 ± 0.2 U/mL) were observed, suggesting that oxidative stress overwhelmed the cellular antioxidant defenses and contributed to mitochondrial dysfunction and subsequent apoptosis. Although GSH depletion in the 20% combination group was moderate rather than profound, apoptosis levels were markedly elevated. This finding suggests that apoptosis induction was not solely dependent on complete GSH exhaustion. Instead, increased lipid peroxidation, mitochondrial dysfunction, and activation of caspase-9-mediated intrinsic apoptotic signaling may have played a more dominant role. Therefore, the high apoptotic response observed in the 20% combination group may reflect a threshold effect in which oxidative imbalance was sufficient to trigger mitochondrial apoptosis despite residual antioxidant capacity.

### 2.9. Limitations of the Study

One limitation of the present study is that the experiments were conducted using only the HeLa cervical cancer cell line. Therefore, further studies using additional cervical cancer models such as SiHa, CaSki, and C-33A cells are required to confirm the reproducibility and broader applicability of these findings. In addition, the absence of normal cell controls limits the evaluation of the selective anticancer potential and safety profile of the combined treatment. Another limitation is that intracellular ROS production was not directly measured using fluorescence-based assays. Oxidative stress was evaluated indirectly through TAS, TOS, OSI, GSH, MDA, and antioxidant enzyme activity analyses. Therefore, future studies including normal cell lines together with direct ROS measurement assays are needed to better clarify the redox-modulating mechanisms and selective anticancer effects of the CDDP + ε-VNF combination.

## 3. Materials and Methods

### 3.1. Materials

Minimal Essential Medium (MEM), Fetal Bovine Serum (FBS), trypsin-EDTA solution, penicillin/streptomycin solution, Dimethyl Sulfoxide (DMSO), CDDP, and ε-VNF were obtained from Sigma-Aldrich (St. Louis, MO, USA). TOS and TAS assay kits were purchased from Mega TIP (Gaziantep, Türkiye). Superoxide Dismutase Assay Kit, Glutathione Assay Kit, and Thiobarbituric Acid Reactive Substances (TBARS) Assay Kits were obtained from Cayman Chemical (Ann Arbor, MI, USA). Caspase-9 Activity Assay kits were purchased from Invitrogen (San Diego, CA, USA). Annexin V-FITC/Propidium Iodide (PI) apoptosis detection kits were obtained from BD Biosciences Pharmingen (San Diego, CA, USA).

### 3.2. Cell Culture

An HeLa cell line obtained from the German Collection of Microorganism and Cell Culture (DSMZ, Leibniz Institute, Braunschweig, Germany). HeLa cells were maintained in MEM supplemented with 10% FBS and 1% penicillin-streptomycin solution. Cells were incubated at 37 °C in a humidified atmosphere containing 5% CO_2_ and 95% air. Cells were routinely monitored microscopically for morphology and contamination, and experiments were conducted using low-passage cultures.

### 3.3. Preparation of Drug Concentrations and Treatment Groups

Stock solutions of CDDP and ε-VNF were prepared in DMSO and stored at −20 °C until use. Before the experiments, fresh working solutions were prepared by diluting the stock solutions in complete cell culture medium. To prevent DMSO-induced cytotoxicity, the final DMSO concentration in all treatments was adjusted to remain below 0.1%. A concentration range of 10, 20, 40, 60, 85, and 100 µM was used for both agents to determine their IC_50_. Untreated HeLa cells, which received no drug or solvent, were used as the control group.

Based on the IC_50_ values obtained from MTT assays, appropriate concentrations of CDDP and ε-VNF were selected for subsequent experiments. Treatment groups included cells treated with CDDP alone, ε-VNF alone, and a combination of both agents. All treatments were applied for 24 h to evaluate their effects on oxidative stress parameters and apoptosis.

### 3.4. Cell Viability Assay (MTT Assay)

The cell viability effects of CDDP, ε-VNF, and their combination on HeLa cells were evaluated using the MTT assay. HeLa cells were seeded into 96-well plates at a density of 8 × 10^3^ cells/well and incubated for 24 h to allow cell attachment. After incubation, cells were treated with various concentrations of CDDP and ε-VNF, individually and in combination, for 24 h. Following treatment, 20 µL of MTT solution (5 mg/mL) was added to each well and incubated for an additional 4 h at 37 °C. After incubation, the supernatant was carefully removed, and 100 µL of DMSO was added to each well to dissolve the formazan crystals. The colorimetric change was measured at 540 nm using an ELx800 BioTek microplate reader (Winooski, VT, USA). The MTT assay was performed in three independent experiments, using five replicate wells per concentration group.

### 3.5. Combined Drug Effect Analysis

For the combination studies, various ratios of CDDP and ε-VNF were prepared based on the IC_50_ values previously determined by the MTT assay. The combinations included 100%, 75%, 50%, 40%, 30%, 20%, and 10% of the IC_50_ concentrations of each agent. The inhibitory effects of these combinations on HeLa cell viability were evaluated using the MTT assay. The synergistic or antagonistic interactions between CDDP and ε-VNF were analyzed using CompuSyn software (version 1.0; ComboSyn Inc., Paramus, NJ, USA), based on the Chou–Talalay method [54]. The CI values were calculated, and a corresponding CI-effect plot was generated to characterize the nature of the drug interactions.

### 3.6. Preparation of Cell Lysates

HeLa cells were seeded into 6-well plates at a density of 7 × 10^5^ cells/well and incubated for 24 h to allow for cell attachment. Following this initial incubation, cells were treated with the IC_50_ concentrations of CDDP, ε-VNF, and their combination, and incubated for an additional 24 h. At the end of the treatment period, the culture medium was aspirated and cells were gently washed with cold phosphate-buffered saline (PBS). Adherent cells were detached using a sterile cell scraper and transferred into falcon tubes. The collected cell suspensions were centrifuged at 1600 rpm for 6 min at 4 °C. The resulting pellet was then homogenized using a mechanical homogenizer. Following homogenization, the samples were centrifuged at 13,000 rpm for 20 min at 4 °C. The obtained supernatants were collected and stored at −20 °C until further biochemical analysis. Prepared cell lysates were used to determine oxidative stress parameters including TOS, TAS, SOD activity, GSH levels, and MDA content.

### 3.7. Determination of TOS, TAS Levels and OSI

#### 3.7.1. Measurement of Total Oxidant Status

The TOS of the samples was determined using a commercially available kit (Rel Assay Diagnostics, Cat. No: KM211360; Mega TIP, Gaziantep, Türkiye) according to the manufacturer’s instructions. This method provides an overall assessment of the total oxidant molecules present in the sample [55]. The assay is based on the oxidation of ferrous ion to ferric ion in the presence of various oxidant species. The ferric ion forms a colored complex with a chromogenic substrate in an acidic medium, and the intensity of this color is measured spectrophotometrically.

Absorbance of the cell lysates, prepared as described previously, was measured at 530 nm using a UV-visible spectrophotometer (Shimadzu UV-16A, Kyoto, Japan). The results were expressed as micromoles of hydrogen peroxide equivalent per liter (µmol H_2_O_2_ equiv./L).

The following formula was used for calculation:

$$TOS\left(µmol/L\right)=\frac{\Delta \text{Sample}\;\text{Absorbance}}{\Delta \text{Standard}\;\text{Absorbance}}\times 10$$

#### 3.7.2. Measurement of Total Antioxidant Status

The TAS of the samples was determined using a commercial kit (Rel Assay Diagnostics, Cat. No: KM21123A; Mega TIP, Gaziantep, Türkiye), in accordance with the manufacturer’s instructions. This method measures the total antioxidant capacity of the sample against free radical species [56]. The assay is based on the ability of antioxidants in the sample to inhibit the oxidation reaction of a specific radical substrate.

Absorbance was measured at 660 nm using a spectrophotometer. Results were expressed as micromoles of Trolox equivalent per liter (µmol Trolox equiv./L).

$$TAS\;(mmol/L)=\frac{\;[\left(\Delta dH20\;\text{Absorbance}\right)-\left(\Delta \text{Sample}\;\text{Absorbance}\right)]}{\left[\left(\Delta dH20\;\text{Absorbance}\right)-(\Delta \text{Standard}\;\text{Absorbance})\right]}$$

#### 3.7.3. Calculation of Oxidative Stress Index

The OSI was calculated using the measured TOS and TAS levels. Since the TAS values were originally expressed in mmol/L, they were converted to µmol/L prior to the OSI calculation. The OSI was calculated using the following formula:

OSI=[TOS/TAS]×100

### 3.8. Determination of Superoxide Dismutase Activity

SOD activity was measured using the Cayman Chemical Superoxide Dismutase Assay Kit (Cat. No: 706002; Ann Arbor, MI, USA), in accordance with the manufacturer’s protocol. The assay is based on a colorimetric method that utilizes a tetrazolium salt to detect superoxide radicals generated by xanthine oxidase and hypoxanthine. SOD activities of the pretreated and lysed samples were determined by ELISA (BioTek ELx800, USA) at 450 nm using a microplate reader. The results were expressed in accordance with the kit’s standard curve.

### 3.9. Determination of Glutathione Levels

GSH levels were measured using the Cayman Chemical Glutathione Assay Kit (Cat. No: 703002; Ann Arbor, MI, USA), following the manufacturer’s instructions. This colorimetric assay quantifies total GSH in biological samples based on the enzymatic recycling method using glutathione reductase. GSH levels in pretreated and lysed samples were determined by ELISA (BioTek ELx800, USA) at an absorbance of 405 nm using a microplate reader. Results were calculated based on the kit’s standard curve.

### 3.10. Determination of Malondialdehyde Levels

MDA levels were measured using the Cayman Chemical TBARS Assay Kit (Cat. No: 10009055; Ann Arbor, MI, USA), in accordance with the manufacturer’s instructions. This assay is based on the reaction between MDA and thiobarbituric acid (TBA) under high temperature and acidic conditions, forming a pink chromogen that can be quantified spectrophotometrically. MDA levels in pretreated and lysed samples were determined by ELISA (BioTek ELx800, USA) at 540 nm using a microplate reader. Results were calculated according to the standard curve provided in the kit.

### 3.11. Determination of Apoptosis by Annexin V-FITC/PI Staining

Apoptotic cell death was assessed using the FITC Annexin V Apoptosis Detection Kit (Cat. No: 556547; BD Biosciences, San Jose, CA, USA), following the manufacturer’s protocol. The assay is based on the binding of Annexin V to phosphatidylserine residues exposed on the outer leaflet of the plasma membrane in early apoptotic cells, and the simultaneous use of propidium iodide (PI) to identify late apoptotic or necrotic cells. After treatment, cells were collected and stained with FITC-conjugated Annexin V and PI, then analyzed by BD Accuri^TM^ C6 flow cytometry (BD Biosciences, San Jose, CA, USA). The percentage of viable, early apoptotic, late apoptotic, and necrotic cells was determined based on fluorescence intensity.

### 3.12. Determination of Active Caspase-9 Activity

The activity of active caspase-9 was determined using the CaspGLOW™ Fluorescein Active Caspase-9 Staining Kit (Cat. No: 88-7006-42; BioVision Inc., Milpitas, CA, USA), in accordance with the manufacturer’s instructions. This method detects active caspase-9 in intact cells via a fluorescein-labeled caspase-specific inhibitor that binds to the enzymatically active form. Following treatment, cells were stained according to the kit protocol, and caspase-9 activity was analyzed by flow cytometry. The fluorescence intensity was used to quantify caspase activation in the experimental groups.

### 3.13. Statistical Analysis

All experiments were performed in triplicate, and the results were expressed as mean ± standard deviation (SD). Statistical analyses were conducted using GraphPad Prism version 8.0.1 (GraphPad Software Inc., San Diego, CA, USA). The significance of differences between groups was assessed using appropriate statistical tests as indicated in the figure legends. Statistical significance was defined as follows: * *p* < 0.05, ** *p* < 0.01, *** *p* < 0.001, **** *p* < 0.0001.

## 4. Conclusions

In conclusion, the combination of ε-VNF with CDDP enhanced ROS-mediated mitochondrial apoptosis in HeLa cells, as evidenced by increased caspase-9 activation, Annexin V/PI staining, and altered oxidative stress markers. The synergistic effects observed at lower combination doses highlight the potential of this strategy to mitigate CDDP-associated toxicity. These findings support the potential role of ε-VNF as an adjuvant to chemotherapy, warranting further investigation through additional studies.

## Figures and Tables

**Table 1:** Viability percentages of HeLa cells after 24 h treatment with various concentrations (10–100 µM) of CDDP and ε-VNF, as determined by the MTT assay. Data are presented as mean ± standard deviation (SD) from three independent experiments. * *p* < 0.05, ** *p* < 0.01, *** *p* < 0.001, compared with the untreated control group.

Concentration	% Viability (CDDP)	% Viability (ε-VNF)
Control (0 µM)	100	100
10 µM	80.7 ± 7.7 *	98.4 ± 2.7
20 µM	52.3 ± 3.7 ***	48.8 ± 4.4 **
40 µM	44.4 ± 4.7 ***	57.0 ± 3.6 *
60 µM	39.2 ± 7.2 ***	45.4 ± 3.4 **
85 µM	37.0 ± 6.0 ***	42.1 ± 3.7 **
100 µM	40.4 ± 5.2 ***	47.5 ± 3.8 **

**Table 2:** TOS and TAS levels measured in HeLa cells following 24 h treatment with CDDP, ε-VNF at IC_50_ doses, and various combinations of both agents. Data are presented as mean ± SD from three independent experiments. * *p* < 0.05, ** *p* < 0.01, *** *p* < 0.001, statistically significant compared to the control group.

Groups	TOS (µmol/L)	TAS (µmol/L)
Control	61.7 ± 9.3	266.7 ± 0.1
CDDP IC_50_	31.7 ± 4.4 ***	666.7 ± 0.1
ε-VNF IC_50_	20.0 ± 5.0 *	1133.0 ± 0.1
40% (CDDP 11.2 µM + ε-VNF 8.4 µM)	35.0 ± 1.7 *	1266.7 ± 0.1
20% (CDDP 5.6 µM + ε-VNF 4.2 µM)	43.3 ± 6.0	2466.7 ± 1.0 *
10% (CDDP 2.8 µM + ε-VNF 2.1 µM)	21.7 ± 4.4 **	1333.3 ± 0.1

**Table 4:** SOD activity levels measured in HeLa cells after 24 h treatment with CDDP, ε-VNF (IC_50_ doses), and various fixed-ratio combinations. Data are presented as mean ± SD from three independent experiments. * *p* < 0.05, statistically significant compared to the control group.

Groups	SOD (U/mL)
Control	1.6 ± 0.1
CDDP IC_50_	0.5 ± 0.1 *
ε-VNF IC_50_	0.4 ± 0.1 *
40% (CDDP 11.2 µM + ε-VNF 8.4 µM)	1.8 ± 0.4
20% (CDDP 5.6 µM + ε-VNF 4.2 µM)	1.1 ± 0.2
10% (CDDP 2.8 µM + ε-VNF 2.1 µM)	0.8 ± 0.1 *

**Table 5:** GSH levels measured in HeLa cells after 24 h treatment with CDDP, ε-VNF (IC_50_ doses), and various fixed-ratio combinations. Data are expressed as mean ± SD from three independent experiments.* *p* < 0.05, ** *p* < 0.01, statistically significant compared to the control group.

Groups	GSH (µM)
Control	1.4 ± 0.1
CDDP IC_50_	0.4 ± 0.2 **
ε-VNF IC_50_	0.6 ± 0.2 *
40% (CDDP 11.2 µM + ε-VNF 8.4 µM)	1.4 ± 0.3
20% (CDDP 5.6 µM + ε-VNF 4.2 µM)	0.8 ± 0.2
10% (CDDP 2.8 µM + ε-VNF 2.1 µM)	0.9 ± 0.1 **

**Table 6:** MDA activation results. MDA levels (µM) in the control, CDDP IC_50_, ε-VNF IC_50_, and combined treatment groups (40%, 20%, and 10%). The results are shown as mean ± SD of three experiments. ** *p* < 0.01, *** *p* < 0.001 when compared with the control.

Groups	MDA (µM)
Control	0.5 ± 0.1
CDDP IC_50_	1.6 ± 0.1
ε-VNF IC_50_	0.9 ± 0.2
40% (CDDP 11.2 µM + ε-VNF 8.4 µM)	4.3 ± 0.6 ***
20% (CDDP 5.6 µM + ε-VNF 4.2 µM)	3.8 ± 0.5 **
10% (CDDP 2.8 µM + ε-VNF 2.1 µM)	3.4 ± 0.4

**Table 7:** Viability, necrosis, and apoptotic cell percentages (early and late) in HeLa cells determined by Annexin V-FITC/PI staining after 24 h treatment with CDDP, ε-VNF, and their combinations. Values are shown as percentages of total cell population.

Groups	Viability (%)	Necrosis (%)	Late Apoptosis (%)	Early Apoptosis (%)
Control	96.9	0.1	1.2	1.9
CDDP IC_50_	1.1	0.8	89.1	8.9
ε-VNF IC_50_	95.6	0.6	2.6	1.2
40% (CDDP 11.2 µM + ε-VNF 8.4 µM)	66.4	2.9	27.4	3.3
20% (CDDP 5.6 µM + ε-VNF 4.2 µM)	5.1	1.8	76.1	17.0
10% (CDDP 2.8 µM + ε-VNF 2.1 µM)	86.0	1.1	10.5	2.5

**Table 8:** Percentage of caspase-9 positive and negative HeLa cells after 24 h treatment with CDDP, ε-VNF, and their combinations, as determined by flow cytometry using a caspase-9 specific staining kit. Values represent the percentage of total cell population.

Groups	Negative Caspase-9 (%)	Positive Caspase-9 (%)
Control	85.6	13.2
CDDP IC_50_	65.1	25.7
ε-VNF IC_50_	83.2	15.1
40% (CDDP 11.2 µM + ε-VNF 8.4 µM)	52.1	42.5
20% (CDDP 5.6 µM + ε-VNF 4.2 µM)	33.3	55.0
10% (CDDP 2.8 µM + ε-VNF 2.1 µM)	33.3	55.3

## Data Availability

The original contributions presented in this study are included in the article. Further inquiries can be directed to the corresponding author.

## Abbreviations

The following abbreviations are used in this manuscript:

CAT	Catalase
CDDP	Cisplatin; cis-diamminedichloroplatinum(II)
CI	Combination Index
DMSO	Dimethyl Sulfoxide
Fa	Fraction affected
FBS	Fetal Bovine Serum
GPx	Glutathione Peroxidase
GSH	Reduced Glutathione
HPV	Human Papillomavirus
IC_50_	Half-maximal inhibitory concentrations
MDA	Malondialdehyde
MEM	Minimal Essential Medium
MTT	3-(4,5-dimethylthiazol-2-yl)-2,5-diphenyltetrazolium bromide
OSI	Oxidative Stress Index
PBS	Phosphate-Buffered Saline
PI	Propidium Iodide
ROS	Reactive Oxygen Species
SD	Standard Deviation
SOD	Superoxide Dismutase
TAS	Total Antioxidant Status
TBA	Thiobarbituric Acid
TBARS	Thiobarbituric Acid Reactive Substances
TOS	Total Oxidant Status
VNF	Viniferin
ε-VNF	ε-viniferin
